# Prevalence of Cigarette Smoking and Nicotine Dependence in Men and Women Residing in Two Provinces in China

**DOI:** 10.3389/fpsyt.2017.00254

**Published:** 2017-12-01

**Authors:** Yunlong Ma, Li Wen, Wenyan Cui, Wenji Yuan, Zhongli Yang, Keran Jiang, Xianzhong Jiang, Meijun Huo, Zilong Sun, Haijun Han, Kunkai Su, Shigui Yang, Thomas J. Payne, Jundong Wang, Ming D. Li

**Affiliations:** ^1^State Key Laboratory for Diagnosis and Treatment of Infectious Diseases, The First Affiliated Hospital, Collaborative Innovation Center for Diagnosis and Treatment of Infectious Diseases, Zhejiang University School of Medicine, Hangzhou, China; ^2^Shanxi Key Laboratory of Environmental Veterinary Medicine, Shanxi Agricultural University, Taigu, China; ^3^ACT Center for Tobacco Treatment, Education and Research, Department of Otolaryngology and Communicative Sciences, University of Mississippi Medical Center, Jackson, MS, United States; ^4^Research Center for Air Pollution and Health, Zhejiang University, Hangzhou, China; ^5^Institute of NeuroImmune Pharmacology, Seton Hall University, South Orange, NJ, United Sates

**Keywords:** Chinese, nicotine dependence, smoking initiation, smoking cessation, respiratory and digestive symptoms

## Abstract

**Aims:**

Although it is known that there is a high smoking prevalence among Chinese, key issues such as social and environmental factors impacting smoking initiation and persistence, the percentage of smokers considered nicotine dependence (ND), and the availability and use of ND treatments have rarely been investigated.

**Methods:**

To address these issues, from 2012 to 2014, we conducted a large-scale study in the Zhejiang and Shanxi provinces of China using the Fagerström Test for Nicotine Dependence and other validated questionnaires.

**Results:**

Of the 17,057 subjects, consisting of 13,476 males and 3,581 females aged 15 years or older, the prevalence of male smoking was 66.1% [95% confidence interval (CI) 65.5%, 66.9%] and that of female smoking was 3.2% (95% CI 3.0%, 3.8%). Among males, 25.8% (95% CI 25.0%, 26.5%) were low-to-moderate ND, and 11.8% (95% CI 11.2%, 12.3%) were high ND (H-ND), persons who have significant difficulty quitting without treatment. The degrees of ND were related to age, extent of education, and annual family income. The social–environmental factors examined conveyed a higher risk for smoking initiation, which is particularly true for the influence of smoking by friends. Furthermore, current smokers had a significantly higher risk of suffering respiratory and digestive symptoms.

**Conclusion:**

These data not only show a high smoking prevalence in Chinese men but also reveal that a relatively large number of smokers are H-ND. Considering that few Chinese smokers seek ND treatment, a comprehensive smoking prevention and treatment program designed specifically for Chinese is greatly needed.

## Introduction

As the largest tobacco-consuming country in the world, the prevalence of cigarette smoking in China remains high, especially in the male population (1). Currently, China is facing an immense public health challenge from tobacco smoking, with about 1.4 million deaths resulting from cigarette smoking in 2010 (2). This number is expected to increase to 2 million by 2030 and 3 million by 2050 if smoking patterns are not altered (3).

The economic burden caused by tobacco smoking in China has increased substantially during the past decade. In 2008, approximately US$6.2 billion was spent for direct smoking-attributable health care costs and US$22.7 billion was spent for indirect economic costs, a rise of 154 and 376%, respectively, in comparison with costs in 2000 (4). Although in 2005, China ratified the treaty of the WHO Framework Convention on Tobacco Control (FCTC) to make progress on smoking prevention and reduction (5), the country is still in the early stage of addressing the threat from cigarette smoking, with significant gaps still present in the response to the requirements of FCTC (6).

Prevention of smoking initiation and promotion of smoking cessation remain important ways of tobacco control (6–8). Although many smokers want to quit, only a few succeed (9–11). Various genetic, social, and psychological factors have been implicated in difficulties achieving and maintaining cessation (12–16). One of the key inhibiting factors is physical dependence on nicotine (17). The epidemic of nicotine dependence (ND) harbors enormous harmful effects on the health of smokers. Accumulating evidence (18–21) demonstrates that time to the first cigarette of the day, one of the best indicators of ND (22), correlates with higher extents of smoking relapse, greater withdrawal symptoms, continued nicotine intake, tobacco-related carcinogen exposure, and cancer risk in both adolescent and adult smokers.

Another important factor preventing abstinence is the social conventions in China, where smoking has long had a positive and attractive image (6, 23). Many Chinese smokers do not fully understand the adverse consequences of tobacco smoking. At almost all social events, such as weddings, funerals, and official activities, cigarettes are considered an essential supply for all attendees, especially in rural areas (6). It is a courtesy to offer cigarettes to friends, guests, or visitors; and in 2010, only 23.2% of Chinese adults believed that cigarette smoking leads to stroke, heart attack, and lung cancer (24). Thus, it is urgent to raise public awareness of the harmful consequences of smoking and to increase the government’s incentives to take steps to reduce the high smoking rate in China.

Although several studies have documented the prevalence of smoking among Chinese adults (1, 9, 23, 25, 26), none has broadly examined the prevalence of ND. To address this issue and provide information that has implications for tobacco control, we conducted a large-scale, cross-sectional study of tobacco use among adults by employing a well-accepted standardized questionnaire. Furthermore, we evaluated the association of social-environmental factors with cigarette smoking, as well as adverse effects of cigarette smoking on health.

## Materials and Methods

### Study Populations

From June 2012 to January 2014, a total of 17,057 persons consisting of 3,581 women and 13,476 men participated in the study. All participants, who were at least 15 years old and visited the hospital for their annual healthy examinations, were invited to participate in the study in their local community hospitals in the southern Zhejiang Province and the northern Shanxi Province of China. We informed each potential participants about the purpose of this study, which was to investigate the epidemic of smoking and ND in Chinese males and females. Demographic characteristics, namely age, sex, education, marital status, annual family income, number of cohabiting family members, smoking status, and medical history, were obtained from each participant, which was conducted as we did in our recruitment of Mid-South Tobacco Family study and Mid-South Tobacco Case–Control Study in the US from 1999 to 2012 (27–30). All subjects who agreed to participate in the study were face-to-face interviewed according to the protocol by local physicians and nurses of each hospital, who were trained by senior investigators of the research team prior to start their involvement of the study. All subjects received a water cup (value about US$2.00) as an appreciation of their participation and time. The Institutional Review Board of the First Affiliated Hospital of Zhejiang University approved the study, and written consent was obtained from all participants.

### Classification of Cigarette Smoking and Dependence on Nicotine

Following the definition of regular smokers commonly used in the tobacco field (31, 32), we defined current smokers as those who had smoked at least 100 cigarettes in their lifetimes and smoked either daily or occasionally at the time of the survey. Former smokers were defined as those who had smoked at least 100 cigarettes but had not smoked for at least 1 year prior to the survey (31, 32). Non-smokers were defined as those who had smoked fewer than 100 cigarettes in their lifetimes. For current and former smokers, we also collected smoking-related information, including age at which they began regular smoking, the location of initial smoking (school, workplace, home, or others), the people who were present at their initial smoking episode (peers, family members, or alone), parent and other family member smoking status, and the percentage of friends who smoked.

Nicotine dependence was evaluated by the Fagerström Test for Nicotine Dependence (FTND) (33) in a Chinese-translated version, which has been documented to be a useful instrument for evaluating ND in Chinese adults (34, 35). To characterize the ND status of each smoker, we categorized smokers into the following three subgroups based on FTND score: very low ND (VL-ND; FTND 0–2), low-to-moderate ND (LM-ND; FTND 3–5), and high ND (H-ND; FTND 6–10) (36), with a goal of achieving an approximately equal sample size of each group.

### Respiratory and Digestive Symptoms

Because of the coal mining in the Shanxi Province where part of the survey was conducted, all participants from this province (*n* = 6,112) were also surveyed for their respiratory and digestive symptoms. We classified participants as individuals with respiratory symptoms according to their answers to the following questions (37): “Did you have any of the following respiratory symptoms in the most recent year?” The options for this question were six kinds of symptoms; i.e., cough, phlegm, chest pain, dyspnea, nasosinusitis, and chronic pharyngitis. Similarly, participants were considered as patients with digestive symptoms according to their answer to the question: “Did you have any of the following digestive symptoms in the most recent year?” There were four kinds of digestive symptoms, namely, nausea, anorexia, acid regurgitation, and stomach cold.

### Statistical Analysis

All data were entered in Epidata 3 independently by two researchers and then double-checked by comparing the entries, with correction of all inconsistent entries before performing any statistical analysis. Differences between proportions among groups were compared for significance with the Chi-square test. Logistic regression analysis was performed to identify the factors associated with smoking and the adverse effect of smoking on the respiratory and digestive systems. All multivariate regression models were adjusted for age, marital status, extent of education, annual family income, and body mass index (BMI), as well as for social-environmental factors. Furthermore, to avoid any potential confounding factor related to respiratory or digestive symptoms, we purposely selected a subgroup of individuals who were free from any respiratory or digestive symptom as a reference control for our analyses. All statistical analyses were conducted with SAS statistical software (version 9.1.3) for Windows (SAS Institute Inc., Cary, NC), and *P* < 0.05 was considered statistically significant.

## Results

### Demographic Characteristics of Participants

There were 17,955 Han Chinese invited to participate the study; of them, 17,057 participants were enrolled into the study. Of all participants, 13,476 (79.0%) were men and 3,581 (21.0%) were women. The participants had an average age of 42.7 years (±12.8 SD) and a mean BMI of 23.8 (±3.2) kg/m^2^, and 87% were married. The proportion of male participants who were college graduates or higher (20.2%) was significantly greater than that of females (16.5%; *P* < 0.0001), which was consistent with the patterns observed in those who attended middle and high school. For detailed information, please refer to Table 1.

**Table 1:** Basic information on participants.

Characteristic	Men	Women	Total
Sample size (%)	13,476 (79.0)	3,581 (21.0)	17,057 (100.0)
Age (years) (%)			
15–24	1,128 (8.4)	138 (3.9)	1,266 (7.4)
25–34	3,094 (23.0)	507 (14.2)	3,601 (21.1)
35–44	3,676 (27.3)	925 (25.9)	4,601 (27.0)
45–54	3,486 (25.9)	1,072 (30.0)	4,558 (26.7)
55–64	1,471 (10.9)	623 (17.5)	2,094 (12.3)
≥65	621 (4.6)	303 (8.5)	924 (5.4)
Mean age (years) (SD)	41.8 (12.7)	48.5 (13.3)	42.7 (12.8)
Mean body mass index (kg/m^2^) (SD)	24.0 (3.2)	22.9 (3.1)	23.8 (3.2)
Marital status (%)			
Married	11,566 (86.0)	3,239 (90.7)	14,805 (87.0)
Never married	1,702 (12.6)	189 (5.3)	1,891 (11.1)
Others	187 (1.4)	143 (4.0)	330 (1.9)
Education (%)			
Elementary school or less	1,802 (13.4)	1,235 (34.9)	3,037 (17.9)
Middle school	5,441 (40.6)	1,160 (32.8)	6,601 (39.0)
High school	3,454 (25.8)	562 (15.9)	4,016 (23.7)
College or beyond	2,709 (20.2)	583 (16.5)	3,292 (19.4)
Annual family income per year (Ұ) (%)			
<20,000	2,011 (15.2)	1,070 (30.8)	3,081 (18.5)
20,001–30,000	1,997 (15.1)	561 (16.2)	2,558 (15.3)
30,001–50,000	2,871 (21.8)	618 (17.8)	3,489 (20.9)
>50,001	6,318 (47.9)	1,223 (35.2)	7,541 (45.2)
Smoking status (%)			
Non-smoker	4,075 (30.2)	3,467 (96.8)	7,542 (44.2)
Current smoker	8,911 (66.1)	113 (3.2)	9,024 (52.9)
Former smoker	490 (3.6)	0	490 (2.9)

*Data may not total 100% because of rounding*.

### Prevalence of Cigarette Smoking and Extent of ND

**Figure 1:**
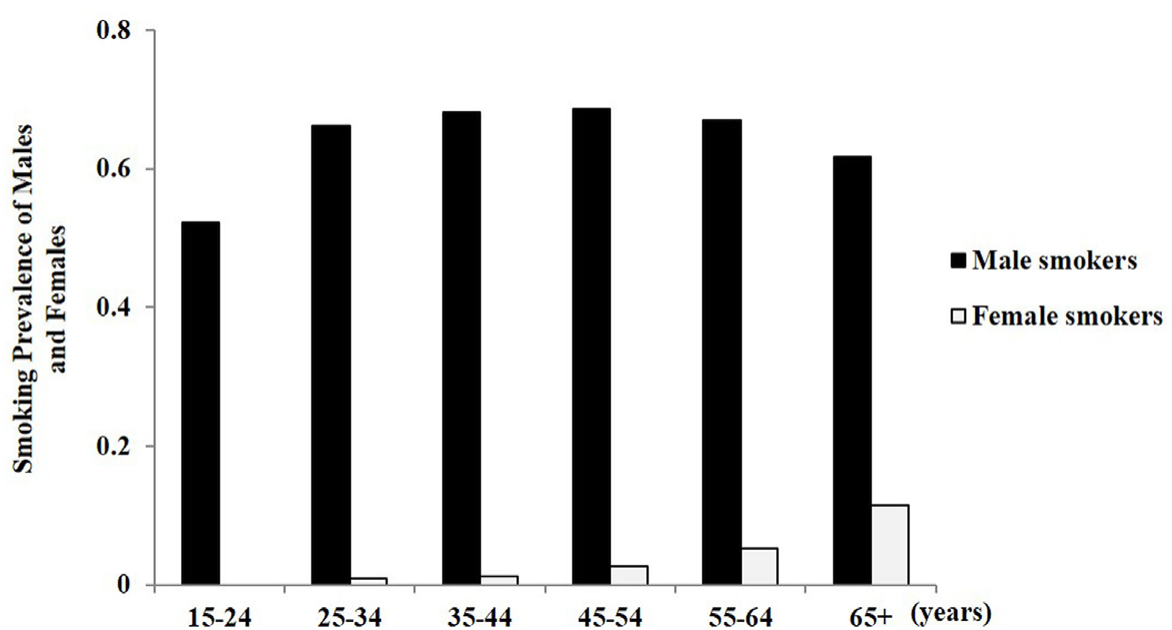
The prevalence of current smoking in male and female adults by age.

The smoking prevalence of participants was 66.1% [95% confidence interval (CI) 65.5%, 66.9%] for men and 3.2% (95% CI 3.0%, 3.8%) for women. For male smokers, the prevalence of current smoking (68.6%) was highest among those aged 45–54 years (95% CI 67.1%, 70.2%) and lowest among those aged 15–24 years (52.3%; 95% CI 49.4%, 55.2%; Table 2). For women, the smoking rate was the highest among those aged 65 years or older (11.5; 95% CI 7.92%; Figure 1).

**Table 2:** Prevalence of current smoking status and degree of nicotine dependence among Chinese male adults.

Characteristics	% Current smokers [95% confidence interval (CI)]	% VL-ND smokers (95% CI)	% LM-ND smokers (95% CI)	% H-ND smokers (95% CI)
All	66.1 (65.5, 66.9)	27.2 (26.4, 27.9)	25.8 (25.0, 26.5)	11.8 (11.2, 12.3)
Age (years)				
15–24	52.3 (49.4, 55.2)	31.6 (28.9, 34.4)	16.4 (14.2, 18.6)	3.63 (2.54, 4.72)
25–34	66.2 (64.5, 67.9)	35.9 (34.2, 37.6)	22.7 (21.2, 24.1)	6.53 (5.66, 7.40)
35–44	68.2 (66.7, 69.7)	28.3 (26.8, 29.7)	26.5 (25.0, 27.9)	12.0 (10.9, 13.0)
45–54	68.6 (67.1, 70.2)	21.9 (20.5, 23.2)	27.0 (25.5, 28.5)	17.6 (16.3, 18.8)
55–64	67.0 (64.6, 69.4)	18.2 (16.2, 20.1)	32.5 (30.1, 34.9)	15.4 (13.6, 17.3)
≥65	61.7 (57.9, 66.9)	20.3 (17.1, 23.5)	31.1 (27.4, 34.7)	10.3 (7.91, 12.7)
Marital status				
Married	67.9 (67.1, 68.8)	26.7 (25.9, 27.5)	26.9 (26.1,27.7)	12.8 (12.2, 13.4)
Never married	54.0 (51.6, 56.4)	31.1 (28.9, 33.3)	17.4 (15.6, 19.2)	4.21 (3.26, 5.17)
Education				
Elementary school or less	68.1 (65.9,70.2)	19.3 (17.4, 21.1)	34.2 (32.0, 36.4)	13.9 (12.3, 15.5)
Middle school	69.5 (68.3, 70.7)	24.9 (23.7, 26.0)	28.2 (27.0, 29.5)	14.7 (13.7, 15.7)
High school	64.9 (63.3, 66.4)	30.6 (29.1, 32.1)	23.1 (21.7, 24.4)	10.2 (9.24, 11.2)
College or beyond	59.6 (57.7, 61.4)	32.1 (30.3, 33.8)	19.2 (17.7, 20.7)	6.79 (5.84, 7.74)
Annual family income (Ұ)				
<20,000	62.4 (60.3, 64.6)	19.7 (17.9, 21.4)	26.1 (24.2, 28.0)	14.3 (12.8, 15.9)
20,001–30,000	65.8 (63.7, 67.9)	21.2 (19.4, 23.0)	28.6 (26.7, 30.6)	13.5 (12.0, 15.0)
30,001–50,000	64.8 (63.0, 66.5)	25.3 (23.7, 26.9)	24.3 (22.7, 25.9)	13.0 (11.7, 14.2)
>50,001	69.4 (68.3, 70.6)	32.7 (31.5, 33.8)	25.6 (24.5, 26.6)	9.71 (8.98, 10.4)

*V-D, very low nicotine dependence; LM-D, low-to-moderate nicotine dependence; H-ND, high nicotine dependence*.

**Figure 2:**
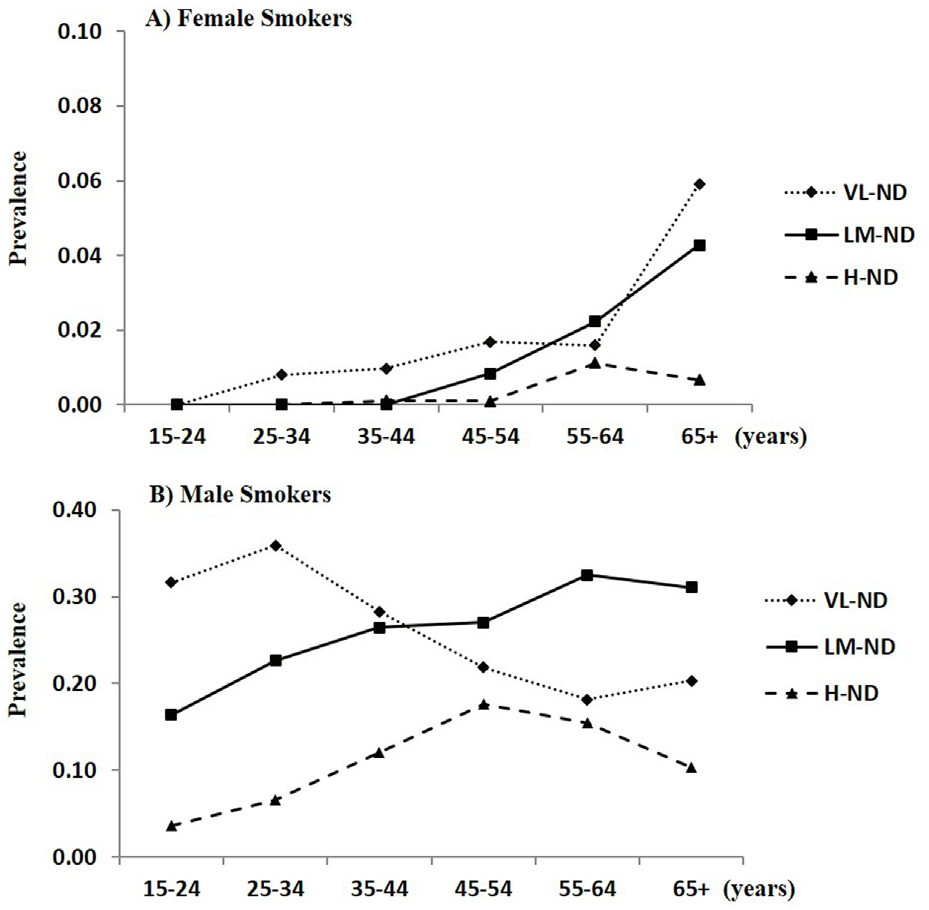
Nicotine dependence prevalence in **(A)** female and **(B)** male adults according to degree of nicotine dependence and age.

Among female smokers, the prevalence of H-ND was 0.31% (95% CI 0.13%, 0.49%), that of LM-ND was 1.01% (95% CI 0.68, 1.34%), and that of VL-ND was 1.66% (95% CI 1.24%, 2.07%). Thus, there was an increasing trend with rising years of age and those aged 65 years or older achieved the highest rates in the LM-ND and VL-ND subgroups (Figure 2A). For male smokers, 11.8% (95% CI 11.2%, 12.3%) were considered to be H-ND, 25.8% (95% CI 25.0%, 26.5%) were considered to be LM-ND, and 27.2% (95% CI 26.4%, 27.9%) were considered to be VL-ND (Table 2). Classified by age, the highest H-ND group was the group 45–54 years old (17.6%; 95% CI 16.3%, 18.8%), while the lowest H-ND group was that 15–24 years old (3.63%; 95% CI 2.54%, 4.72%). For LM-ND, those aged 55–64 years showed the highest percentage (32.5%; 95% CI 30.1%, 34.9%), and the lowest percentage was among those aged 15–24 years (16.4%, 95% CI 14.2%, 18.6%). VL-ND was reported most frequently among those aged 25–34 years (35.9%, 95% CI 34.2%, 37.6%). The prevalence of VL-ND decreased with increasing age, and the prevalence of H-ND was especially higher among those aged 65 years or older compared to other age groups (Figure 2B).

In terms of education (Table 2), the prevalence of smoking was the highest among males who attended only middle school (69.5%; 95% CI 68.3%, 70.7%) and lowest among those who were college graduates or more (59.6%; 95% CI 57.7%, 61.4%). H-ND was common among those who either attended elementary school or less (13.9%; 95% CI 12.3%, 15.5%) or only middle school (14.7%; 95% CI 13.7%, 15.7%); these two groups also demonstrated the lowest proportion of VL-ND (19.3%; 95% CI 17.4, 21.1, and 24.9%; 95% CI 23.7%, 26.0%, respectively). For the family income of male subjects (Table 2), those who earned at least Ұ 50,001 annually had the highest prevalence of smoking (69.4%; 95% CI 68.3%, 70.6%) and VL-ND (32.7%; 95% CI 31.5%, 33.8%), whereas this group had the lowest prevalence of H-ND (9.71%; 95% CI 8.98%, 10.4%). LM-ND was relatively evenly distributed across income categories.

For all male current smokers, an average of 14.5 ± 9.1 cigarettes was smoked each day (Table 3). Those aged 55–64 years smoked 19.9 ± 10.0 cigarettes per day (CPD). In the same age group, those who were H-ND smoked an average of 26.7 ± 12.4 CPD (Table S1 in Supplementary Material). As for all female current smokers, an average of 14.4 ± 7.9 cigarettes was smoked per day, and again, those 65 years of age or older had the highest average CPD (17.3 ± 8.1; Table 3).

**Table 3:** Cigarettes smoked per day (CPD) by male and female smokers according to age.

Characteristic	Age group (years)						Total
	15–24	25–34	35–44	45–54	55–64	>65	
Number of male smokers	587	2,037	2,500	2,386	990	383	8,883
CPD ± SD	7.6 ± 5.2	10.4 ± 6.5	13.8 ± 8.2	17.6 ± 9.8	19.9 ± 10.0	19.0 ± 7.2	14.5 ± 9.1
Number of female smokers	0	4	10	28	31	33	106
CPD ± SD	–	4 ± 4.1	8.4 ± 5.5	12.8 ± 7.6	16.1 ± 6.9	17.3 ± 8.1	14.4 ± 7.9

### Factors Associated with Smoking Initiation

Our data showed that 70.5% (95% CI 69.6%, 71.5%) of male smokers started to smoke daily at the age of 18–25 years (Table 4), whereas 67.0% (95% CI 58.0%–75.9%) of female smokers started at 26 years or older. Male smokers who first smoked at school or in the workplace had the highest proportion of continued smokers, 58.0% (95% CI 56.9%, 59.1%), in contrast to the 65.0% (95% CI 54.6%, 75.5%) of female smokers who first smoked at home. There was an estimated 81.9% (95% CI 81.1%, 82.8%) of male smokers and 64.0% (95% CI 54.6%, 73.4%) of female smokers who first smoked with their friends, whereas only 13.7% (95% CI 12.9%, 14.5%) of male smokers and 22.0% (95% CI 13.9%, 30.1%) of female smokers first smoked alone.

**Table 4:** Characteristics of first tobacco use by male and female current smokers.

Characteristics	Male % [95% confidence interval (CI)]	Female % (95% CI)
Age began regular smoking (years)	*N* = 8,851	*N* = 106
≤17	13.2 (12.4, 13.9)	3.77 (0.15, 7.40)
18–25	70.5 (69.6, 71.5)	29.3 (20.6, 37.9)
≥26	16.3 (15.6, 17.1)	67.0 (58.0, 75.9)
Location of initial smoking	*N* = 7,355	*N* = 80
School/workplace	58.0 (56.9, 59.1)	25.0 (15.5, 34.5)
Home	28.2 (27.1, 29.2)	65.0 (54.6, 75.5)
Others	13.8 (13.0, 14.6)	10.0 (3.43, 16.6)
Others present at initial smoking	*N* = 7,983	*N* = 100
Peers	81.9 (81.1, 82.8)	64.0 (54.6, 73.4)
Alone	13.7 (12.9, 14.5)	22.0 (13.9, 30.1)
Family members	4.4 (03.9, 04.8)	14.0 (7.20, 20.8)

Social-environmental factors exerted vital effects on the initiation of smoking for both sexes (Table 5; Table S2 in Supplementary Material). Those whose parents smoked were more likely to smoke themselves [males: adjusted odds ratio (OR) 1.43; 95% CI 1.30, 1.57; *P* < 0.0001; females: adjusted OR 1.86; 95% CI 1.05, 3.31; *P* = 0.03]. The number of smokers who lived in the household probably conferred a greater risk of smoking for adults, especially for males (number of smokers living in the household ≥2; adjusted OR 1.58; 95% CI 1.40, 1.79; *P* < 0.0001). Also, the percent of friends who smoked was highly correlated with the risk of smoking. Compared with those for whom 25% or less of their friends smoked, those male or female individuals with approximately 100% smoker friends had about a fivefold higher risk of smoking (Table 5; Table S2 in Supplementary Material).

**Table 5:** Influence of selected social-environmental factors on smoking status of male adults.

Characteristic	Number of non-smokers	Number of current smokers	Unadjusted odds ratio (OR) [95% confidence interval (CI)]	*P* value	Adjusted OR^a^ (95% CI)	*P* value
**Parents smoking**						
No	1,224	1,841	1		1	
Yes	2,707	6,868	1.69 (1.55, 1.84)	<0.0001	1.43 (1.30, 1.57)	<0.0001

**Number of smokers living in household**						
0	1,936	3,478	1		1	
1	1,566	3,429	1.22 (1.12, 1.32)	<0.0001	1.13 (1.03, 1.24)	0.01
≥2	545	1,971	2.01 (1.80, 2.25)	<0.0001	1.58 (1.40, 1.79)	<0.0001

**Percentage of smoking friends**						
≤25	1,233	1,121	1		1	
26–50	1,301	2,412	2.04 (1.84, 2.27)	<0.0001	2.00 (1.79, 2.24)	<0.0001
51–75	1,131	3,744	3.64 (3.28, 4.04)	<0.0001	3.57 (3.18, 3.99)	<0.0001
≥76	344	1,609	5.15 (4.47, 5.93)	<0.0001	5.11 (4.40, 5.94)	<0.0001

*^a^Adjusted for age, marital status, education, annual family income, and body mass index, as well as for social-environmental factors using multiple logistic regression models*.

### The Patterns of Smoking Cessation

Of the surveyed participants, only 2.9% were classified as former smokers (3.6% of men and no women) (Table 1). This indicates that very few smokers made any effort to quit or were successful at quitting. Among all male current smokers, those who had quit smoking at some time but were currently smoking accounted for 32.7% (Table 6). Of those who experienced smoking relapse, 90.1% did not receive any treatment or counseling on smoking cessation and only 9.9% quit smoking by using a nicotine patch, acupuncture, or other method. Similarly, the pattern of methods used to quit smoking was similar among male former smokers and female current smokers (Table 6).

**Table 6:** Patterns of quitting smoking among male and female ever-smokers (current and former).

Questions	Male current smokers (%)	Male former smokers (%)	Female current smokers (%)
Have you ever tried to quit smoking?	2,916 (32.7)	490 (100.0)	26 (31.3)
**Methods used**			
Just quit/stopped suddenly	1,632 (64.8)	317 (81.3)	10 (50.0)
Gradually decreased the number of cigarettes	637 (25.3)	66 (16.9)	9 (45.0)
Products such as nicotine chewing gum/patch	47 (1.87)	2 (0.51)	0
Other methods such as cessation counseling by phone/acupuncture	201 (7.99)	5 (1.28)	1 (5.00)

*There were no female former smokers*.

### Clinical Outcome Attributable to Cigarette Smoking

Under both univariate and multivariate models, logistic regression analyses were carried out to determine the adverse effect of smoking on respiratory and digestive symptoms. Smoking was significantly associated with all respiratory symptoms (adjusted OR 1.42; 95% CI 1.25, 1.61; *P* < 0.0001; Table 7). Cigarette smoking showed a significant association with cough (adjusted OR 1.48; 95% CI 1.29, 1.70; *P* < 0.0001), phlegm (adjusted OR 1.70; 95% CI 1.45, 1.99; *P* < 0.0001), chest pain (adjusted OR 1.55; 95% CI 1.02, 2.35; *P* = 0.04), nasosinusitis (adjusted OR 1.38; 95% CI 1.03, 1.88; *P* = 0.03), and chronic pharyngitis (adjusted OR 1.55; 95% CI 1.11, 2.15; *P* = 0.02). There also was evidence that cigarette smoking increased the risk of dyspnea (adjusted OR 1.52; 95% CI 0.98, 2.36; *P* = 0.06).

**Table 7:** Relation between respiratory symptoms and smoking status among male adults.

Respiratory symptom	Non-smokers (%)	Current smokers (%)	Crude odds ratio (OR) [95% confidence interval (CI)]	*P* value	Adjusted OR^a^ (95% CI)	*P* value
Any	601 (40.1)	1,874 (48.9)	1.43 (1.27, 1.62)	<0.0001	1.42 (1.25, 1.61)	<0.0001
Cough	477 (34.7)	1,563 (44.4)	1.50 (1.32, 1.71)	<0.0001	1.48 (1.29,1.70)	<0.0001
Phlegm	291 (24.5)	1,072 (35.4)	1.69 (1.45, 1.97)	<0.0001	1.70 (1.45, 1.99)	<0.0001
Chest pain	33 (3.5)	111(5.4)	1.54 (1.04, 2.30)	0.03	1.55 (1.02, 2.35)	0.04
Dyspnea	33 (2.6)	115 (5.5)	1.89 (1.24, 2.87)	0.003	1.52 (0.98, 2.36)	0.06
Nasosinusitis	76 (7.8)	197 (9.1)	1.19 (0.90, 1.57)	0.22	1.38 (1.03, 1.88)	0.03
Chronic pharyngitis	53 (5.6)	179 (8.4)	1.65 (1.24, 2.21)	0.0007	1.55 (1.11, 2.15)	0.02

*^a^Adjusted for age, marital status, education, annual family income, and body mass index, as well as for social-environmental factors using multiple logistic regression models*.

With respect to digestive symptoms (Table 8), we found a significant association of smoking with nausea (adjusted OR 1.43; 95% CI 1.12, 1.83; *P* = 0.005) and acid regurgitation (adjusted OR 1.30; 95% CI 1.08, 1.58; *P* = 0.007). Although it was not significant, there was a consistent trend of association of smoking with any symptom (adjusted OR 1.09; 95% CI 0.95, 1.25; *P* = 0.21), anorexia (adjusted OR 1.30; 95% CI 0.92, 1.84; *P* = 0.14), and stomach cold (adjusted OR 1.18; 95% CI 0.92, 1.51; *P* = 0.18). Together, our results show that current smoking might convey a higher risk of both respiratory and digestive symptoms.

**Table 8:** Relation between digestive symptoms and smoking status among male adults.

Digestive symptom	Non-smokers (%)	Current smokers (%)	Crude odds ratio (OR) [95% confidence interval (CI)]	*P* value	Adjusted OR^a^ (95% CI)	*P* value
Any symptom	454 (29.4)	1,294 (33.0)	1.18 (1.04, 1.34)	0.01	1.09 (0.95, 1.25)	0.21
Nausea	97 (8.2)	364 (12.2)	1.56 (1.23, 1.97)	0.0002	1.43 (1.12, 1.83)	0.005
Anorexia	49 (4.3)	153 (5.5)	1.30 (0.93, 1.80)	0.12	1.30 (0.92, 1.84)	0.14
Acid regurgitation	171 (13.6)	623 (19.2)	1.51 (1.26, 1.81)	<0.0001	1.30 (1.08, 1.58)	0.007
Stomach cold	101 (8.5)	327 (11.1)	1.34 (1.06, 1.70)	0.01	1.18 (0.92, 1.51)	0.18

*^a^Adjusted for age, martial status, education, annual family income, and body mass index, as well as for social-environmental factors by multiple logistic regression models*.

## Discussion

In the present study, we provide further evidence of a high prevalence of smoking among Chinese male adults (66.1%) and a low prevalence in female adults (3.2%), which is in accordance with the findings from a recent study (3). Importantly, we first report the ND pattern in China based on a large-scale population. The average FTND score was 3.20 (95% CI 3.15, 3.25) among all Chinese male smokers, which is much higher than that in Chinese city residents (2.89; 95% CI 2.77, 3.01) (34) and slightly lower than that in Chinese rural–urban migrants (3.39; 95% CI 3.24, 3.54) (35). Of all male current smokers, 18.2% were categorized as H-ND and 39.8% were categorized as LM-ND; these smokers typically have considerable difficulty quitting tobacco without medical assistance.

The prevalence of LM-HD and H-ND was higher in old participants (>65 years) than in young ones, which is consistent with previous studies (34, 35). This indicates that psychological and pharmacological treatments should be focused on older smokers. The majority of young smokers were “social” smokers, with L-ND. Furthermore, male Chinese smokers who had low income or poor education were more likely to be H-ND, which concurs with the findings in smokers in other countries, including the USA (38, 39). In low- and middle-income countries, a 10% increase in the price can decrease the demand for cigarettes by 2–8% (40). This indicates that raising the tax on cigarette products will reduce the number of Chinese smokers who are not highly addicted to nicotine.

In the current study, most Chinese male smokers began regular smoking in early adult life. Previous study has documented that in China, approximately two-thirds of young men became cigarette smokers in their early adulthood and more than 6 million young men start smoking annually (3). Although the smoking rate of women is associated with older ages and remains low, it is necessary to maintain this low rate, which is increasing rapidly as the attitude of women toward smoking becomes more favorable (7, 41), as previously seen in central European and Latin American countries (42, 43). Young Chinese women are being targeted by the tobacco industry with the goal of increasing sales (7, 44), a technique that has been applied successfully in other countries (45). A recent meta-analysis showed that the prevalence of current smoking among adolescent Chinese women in 2006–2010 had increased 11.2-fold over 1981–1985 (46). Thus, the Chinese government not only needs to encourage the smoking cessation aggressively but also to protect non-smokers assiduously, especially adolescent and young adults, from smoking initiation (7, 8).

Our findings also demonstrated that social-environmental factors have an effect on initiation of tobacco smoking for both men and women in China. The smoking behaviors of parents, other family members, and friends were significantly associated with the initiation of smoking in the participants. Particularly, most of the male and female smokers were with their peers or friends when they first smoked. Individuals almost all of whose friends smoke had approximately a fivefold higher risk of smoking than those with very few friends who smoked, which is highly correlated with the cultural norms of China, where cigarettes are a requisite is part of a wide range of social events (6, 23).

Although there are a tremendous number of smokers in China, the quit rate is substantially lower than that in the developed countries, such as the UK and USA (6, 9, 10). A positive sign was that a growing number of Chinese smokers intended to quit in 2010 compared to 1996. However, it has been reported that the relapse rate increased from 12% in 1996 to 33% in 2010 (1, 9–11), which is similar to our findings in this study (32.7%). Most male and female smokers who experienced relapse did not use any method to assist with abstinence, and only a small portion of male and female smokers sought professional assistance in quitting. This indicates that offering effective cessation treatments will contribute greatly to a reduction in the prevalence of smoking. Furthermore, our results showed that current smoking remarkably increased the risk of respiratory and digestive symptoms. If nothing is done toward reducing or quitting smoking, these kinds of symptoms probably will develop into deleterious conditions (47, 48), which not only reduces the quality of life of patients themselves but also increases the burden on their families and, indeed, the whole society.

Although China signed the FCTC in 2003 and ratified it 2 years later, the Chinese government seems to be less effective in enforcing it (49). In many regions of China, smoking is still very prevalent; for example, people can smoke freely in a hospital, factory, school, or other public places (41, 49, 50). In addition, there is a very high prevalence of second-hand smoking in China because of relatively poor housing conditions, overcrowding, and poor ventilation systems (51). Data from the China Global Adult Tobacco Survey reported that approximately 740 million non-smokers suffered from second-hand smoking in 2010 (24). Urgently, firm actions should be taken on tobacco control issues. A successful model for China is in Hong Kong, a Special Administrative Region of China, which has sought to diminish the health burden of cigarette smoking for more than 20 years (52). Greater efforts are warranted to increase public awareness of the harmful consequences of cigarette smoking, raise taxes on tobacco products, restrain smoking in public places (e.g., school, work places, and restaurants), require large and graphic health warnings on cigarette packages, discourage social conventions that promote use (e.g., offering cigarettes to others), and establish effective smoking cessation treatments for those requiring assistance. All of these are necessary, as well as extending the medical insurance system to cover the cost of treatments.

Several limitations in this study warrant comments. First, the data used were based on self-report, which might lead to reporting inaccuracy, although it is commonly used in many population-based surveys, including tobacco research. Smoking by women is socially unacceptable in some countries, and in these places, estimates of the prevalence of smoking that depend on self-reporting might hide the true prevalence (9, 53). Second, we investigated the prevalence of smoking and ND in only two provinces of China (Zhejiang and Shanxi provinces), which might be not widely representative of the whole population of the country. However, the smoking pattern in these two provinces probably reflects that in many southern and northern provinces of China. In addition, this was a cross-sectional design-based survey, so temporal associations cannot be inferred. Large-scale longitude-based studies for surveying the pattern of ND in China are warranted.

In conclusion, this study was based on a large sample used to investigate the prevalence of cigarette smoking and the degree of ND in China. The prevalence of male smoking in the country is high, and a great number of smokers are highly dependent on nicotine, a group that has significant difficulties quitting without treatment. Chinese smokers with a considerable interest in cessation are impeded by ND and social-environmental factors. Our results will contribute to understanding the patterns of tobacco smoking in China and to defining the clinical implications for ND treatments. If the present course remains unchanged, in the near future, China will face a challenge of epidemic proportions caused by smoking.

## Ethics Statement

This study was carried out in accordance with the recommendations of the Institutional Review Board guideline of the First Affiliated Hospital of Zhejiang University with written informed consent from all subjects. All subjects gave written informed consent in accordance with the Declaration of Helsinki. The protocol was approved by the Institutional Review Board of the First Affiliated Hospital of Zhejiang University.

## Author Contributions

MDL, JW conceived the study and wrote the article. YM collected the data, performed the data analysis, and wrote the article. LW collected the data and performed the data analysis. WC, WY, ZY, KJ, XJ, MH, ZS, HH, KS, and SY collected the data. TJP wrote the article. All authors read the manuscript and approved.

## Conflict of Interest Statement

The authors declare that the research was conducted in the absence of any commercial or financial relationships that could be construed as a potential conflict of interest.

## References

[1] Li Q Hsia J Yang G Prevalence of smoking in China in 2010. N Engl J Med (2011) 364:2469–70. 10.1056/NEJMc1102459 21696322

[2] Yang G Wang Y Zeng Y Gao GF Liang X Zhou M Rapid health transition in China, 1990-2010: findings from the Global Burden of Disease Study 2010. Lancet (2013) 381:1987–2015. 10.1016/S0140-6736(13)61097-1 23746901 PMC7159289

[3] Chen Z Peto R Zhou M Iona A Smith M Yang L Contrasting male and female trends in tobacco-attributed mortality in China: evidence from successive nationwide prospective cohort studies. Lancet (2015) 386:1447–56. 10.1016/S0140-6736(15)00340-2 26466050 PMC4691901

[4] Yang L Sung HY Mao Z Hu TW Rao K. Economic costs attributable to smoking in China: update and an 8-year comparison, 2000-2008. Tob Control (2011) 20:266–72. 10.1136/tc.2010.042028 21339491 PMC3759015

[5] China. TCPsGotPsRo. Tobacco Control for 5 Years. (2012). Available from: http://www.gov.cn/fwxx/content_1780112.htm

[6] Yang G Wang Y Wu Y Yang J Wan X. The road to effective tobacco control in China. Lancet (2015) 385:1019–28. 10.1016/S0140-6736(15)60174-X 25784349

[7] Koplan J Eriksen M Smoking cessation for Chinese men and prevention for women. Lancet (2015) 386:1422–3. 10.1016/S0140-6736(15)00416-X 26466027

[8] Zhu C Young-soo S Beaglehole R Tobacco control in China: small steps towards a giant leap. Lancet (2012) 379:779–80. 10.1016/S0140-6736(11)61933-8 22386013

[9] Giovino GA Mirza SA Samet JM Gupta PC Jarvis MJ Bhala N Tobacco use in 3 billion individuals from 16 countries: an analysis of nationally representative cross-sectional household surveys. Lancet (2012) 380:668–79. 10.1016/S0140-6736(12)61085-X 22901888

[10] Yang G Global Adult Tobacco Survey (GATS) China 2010 Country Report. Beijing: China Three Gorges Publishing House (2011).

[11] Yang G Fan L Huang Z Li F Chen A Becker K Smoking and Health in China: 1996 National Prevalence Survey of Smoking Pattern. Beijing: Chinese Science and Technology Press (1997).

[12] Tyas SL Pederson LL. Psychosocial factors related to adolescent smoking: a critical review of the literature. Tob Control (1998) 7:409–20. 10.1136/tc.7.4.409 10093176 PMC1751465

[13] Ma Y Wang M Yuan W Su K Li MD. The significant association of Taq1A genotypes in DRD2/ANKK1 with smoking cessation in a large-scale meta-analysis of Caucasian populations. Transl Psychiatry (2015) 5:e686. 10.1038/tp.2015.176 26624925 PMC5068580

[14] Ma Y Yuan W Cui W Li MD Meta-analysis reveals significant association of 3’-UTR VNTR in SLC6A3 with smoking cessation in Caucasian populations. Pharmacogenomics J (2016) 16:10–7. 10.1038/tpj.2015.44 26149737 PMC4705003

[15] Ma Y Yuan W Jiang X Cui WY Li MD. Updated findings of the association and functional studies of DRD2/ANKK1 variants with addictions. Mol Neurobiol (2015) 51:281–99. 10.1007/s12035-014-8826-2 25139281

[16] Li MD Burmeister M. New insights into the genetics of addiction. Nat Rev Genet (2009) 10:225–31. 10.1038/nrg2536 19238175 PMC2879628

[17] Breslau N Johnson EO Hiripi E Kessler R. Nicotine dependence in the United States: prevalence, trends, and smoking persistence. Arch Gen Psychiatry (2001) 58:810–6. 10.1001/archpsyc.58.9.810 11545662

[18] Branstetter SA Muscat JE Time to first cigarette and serum cotinine levels in adolescent smokers: National Health and Nutrition Examination Survey, 2007–2010. Nicotine Tob Res (2013) 15:701–7. 10.1093/ntr/nts189 22990214

[19] Mercincavage M Branstetter SA Muscat JE Horn KA Time to first cigarette predicts cessation outcomes in adolescent smokers. Nicotine Tob Res (2013) 15:1996–2004. 10.1093/ntr/ntt087 23811009 PMC4318927

[20] Baker TB Piper ME McCarthy DE Bolt DM Smith SS Kim S-Y Time to first cigarette in the morning as an index of ability to quit smoking: implications for nicotine dependence. Nicotine Tob Res (2007) 9:S555–70. 10.1080/14622200701673480 18067032 PMC2933747

[21] Branstetter SA Mercincavage M Muscat JE Predictors of the nicotine dependence behavior time to the first cigarette in a multiracial cohort. Nicotine Tob Res (2015) 17:819–24. 10.1093/ntr/ntu236 25431372 PMC4481692

[22] Fagerstrom K Time to first cigarette; the best single indicator of tobacco dependence? Monaldi Arch Chest Dis (2003) 59:91–4. 14533289

[23] Zhang J OU JX BAI CX. Tobacco smoking in China: prevalence, disease burden, challenges and future strategies. Respirology (2011) 16:1165–72. 10.1111/j.1440-1843.2011.02062.x 21910781

[24] Prevention CCfDCa. Global Adult Tobacco Survey (GATS) Fact Sheet China. (2010). Available from: tobacco/global/gats/countries/wpr/fact_sheets/china/2010/indexhtm.

[25] Gu D Wu X Reynolds K Duan X Xin X Reynolds RF Cigarette smoking and exposure to environmental tobacco smoke in China: the international collaborative study of cardiovascular disease in Asia. Am J Public Health (2004) 94:1972–6. 10.2105/AJPH.94.11.1972 15514239 PMC1448571

[26] Weng X Hong Z Chen D Smoking prevalence in Chinese aged 15 and above. Chin Med J (1987) 100:886. 3130228

[27] Li MD Beuten J Ma JZ Payne TJ Lou XY Garcia V Ethnic- and gender-specific association of the nicotinic acetylcholine receptor alpha4 subunit gene (CHRNA4) with nicotine dependence. Hum Mol Genet (2005) 14:1211–9. 10.1093/hmg/ddi132 15790597

[28] Li MD Sun D Lou XY Beuten J Payne TJ Ma JZ. Linkage and association studies in African- and Caucasian-American populations demonstrate that SHC3 is a novel susceptibility locus for nicotine dependence. Mol Psychiatry (2007) 12:462–73. 10.1038/sj.mp.4001933 17179996

[29] Li MD Payne TJ Ma JZ Lou XY Zhang D Dupont RT A genomewide search finds major susceptibility Loci for nicotine dependence on chromosome 10 in African Americans. Am J Hum Genet (2006) 79:745–51. 10.1086/508208 16960812 PMC1592559

[30] Yang J Wang S Yang Z Hodgkinson CA Iarikova P Ma JZ The contribution of rare and common variants in 30 genes to risk nicotine dependence. Mol Psychiatry (2015) 20:1467–78. 10.1038/mp.2014.156 25450229 PMC4452458

[31] CDC. Cirarette smoking among adults – United Sates, 2000. MMWR Morb Mortal Wkly Rep (2002) 51:642–5. 12186222

[32] CDC. Cigarette smoking among adults – United States, 2006. MMWR Morb Mortal Wkly Rep (2007) 56:1157–61. 17989644

[33] Heatherton TF Kozlowski LT Frecker RC Fagerstrom KO The fagerstrom test for nicotine dependence: a revision of the Fagerstrom Tolerance Questionnaire. Br J Addict (1991) 86:1119–27. 10.1111/j.1360-0443.1991.tb01879.x 1932883

[34] Yang T Shiffman S Rockett IR Cui X Cao R. Nicotine dependence among Chinese city dwellers: a population-based cross-sectional study. Nicotine & Tobacco Research (2011) 13:556–64. 10.1093/ntr/ntr040 21454911

[35] Wu J Yang T Rockett IR Xing R Karalic S Li Y Nicotine dependence among rural-urban migrants in China. BMC Public Health (2011) 11:1 10.1186/1471-2458-11-296 21569258 PMC3120682

[36] Fagerstrom KO Heatherton TF Kozlowski L Nicotine addiction and its assessment. Ear Nose Throat J (1990) 69:763–5. 2276350

[37] Higgins IT Whittaker D Chronic Respiratory Disease in Coal Miners: Follow Up Study of Two Mining Communities in West Virginia. Chronic Respiratory Disease in Coal Miners: Follow Up Study of Two Mining Communities in West Virginia. Morgantown, West VA: Department of Health and Human Services, NIOSH (1981).

[38] Kendler KS Neale M Sullivan P Corey L Gardner C Prescott C. A population-based twin study in women of smoking initiation and nicotine dependence. Psychol Med (1999) 29:299–308. 10.1017/S0033291798008022 10218922

[39] Ziedonis DM Kosten TR Glazer WM Frances RJ Nicotine dependence and schizophrenia. Psychiatr Serv (1994) 45:204–6. 10.1176/ps.45.3.204 7910577

[40] Lancet T Tobacco killing in low-income and middle-income countries. Lancet (2012) 379:1172 10.1016/S0140-6736(12)60492-9 22464371

[41] Chen MH Economic concerns hamper tobacco control in China. Lancet (2007) 370:729–30. 10.1016/S0140-6736(07)61359-2 17806145

[42] Lopez AD Collishaw NE Piha T A descriptive model of the cigarette epidemic in developed countries. Tob Control (1994) 3:242 10.1136/tc.3.3.242

[43] Shafey O Dolwick S Guindon G Tobacco Control Country Profiles 2003. Atlanta: American Cancer Society (2003). Consumo de tabaco en médicos residentes de pediatría en la Argentina Prevalencia actual y tendencia en los últimos diez años. 321.

[44] Wright AA Katz IT Tobacco tightrope—balancing disease prevention and economic development in China. N Engl J Med (2007) 356:1493–6. 10.1056/NEJMp078018 17429080

[45] Tye L The Father of Spin: Edward L. Bernays and the Birth of Public Relations. New York: Crown Publishers, Inc. (1998) p. 306.

[46] Han J Chen X A meta-analysis of cigarette smoking prevalence among adolescents in China: 1981–2010. Int J Environ Res Public Health (2015) 12:4617–30. 10.3390/ijerph120504617 25922989 PMC4454929

[47] Lundback B Nystrom L Rosenhall L Stjernberg N. Obstructive lung disease in northern Sweden: respiratory symptoms assessed in a postal survey. Eur Respir J (1991) 4:257–66. 1864340

[48] Enright PL Kronmal R Higgins MW Schenker MB Haponik EF Group CHSR Prevalence and correlates of respiratory symptoms and disease in the elderly. Chest (1994) 106:827–34. 10.1378/chest.106.3.827 8082366

[49] Lancet T China’s unhealthy relations with big tobacco. Lancet (2011) 377:180 10.1016/S0140-6736(11)60028-7 21237385

[50] Stillman F Navas-Acien A Ma J Ma S Avila-Tang E Breysse P Second-hand tobacco smoke in public places in urban and rural China. Tob Control (2007) 16:229–34. 10.1136/tc.2006.018333 17652237 PMC2598539

[51] Wang C Ma S Xu X Wang J Mei C Yang G The prevalence of household second-hand smoke exposure and its correlated factors in six counties of China. Tob Control (2009) 18:121–6. 10.1136/tc.2008.024836 19131456 PMC2655043

[52] Koplan JP An WK Lam RM Hong Kong: a model of successful tobacco control in China. Lancet (2010) 375:1330–1. 10.1016/S0140-6736(10)60398-4 20347129

[53] Jung-Choi K-H Khang Y-H Cho H-J. Hidden female smokers in Asia: a comparison of self-reported with cotinine-verified smoking prevalence rates in representative national data from an Asian population. Tob Control (2012) 21:536–42. 10.1136/tobaccocontrol-2011-050012 21972062

