# Supplementary material for: Prevalence of Cigarette Smoking and Nicotine Dependence in Men and Women Residing in Two Provinces in China
Source: Front Psychiatry. 2017 Dec 1;8:254. doi: 10.3389/fpsyt.2017.00254 (PMC5716983; doi:10.3389/fpsyt.2017.00254)
Supplement: Supplementary file 1 [file Table_1.PDF]

Supplemental Table 1: The statistics of cigarette smoked per day (CPD) among male smokers according to nicotine dependence and age

Characteristics	Age group						Total
	15-24 yr	25-34 yr	35-44 yr	45-54 yr	55-64 yr	>65 yr	
No. of VL-ND smokers	357	1,110	1,039	762	267	126	3,661
CPD (±SD)	5.6 (±3.8)	7.6 (±4.7)	9.2 (±6.0)	11.7 (±6.8)	13.7 (±6.7)	15.0 (±6.0)	9.4 (±6.2)
No. of LM-ND smokers	185	701	973	942	478	193	3,472
CPD (±SD)	10.1 (±5.2)	12.6 (±6.3)	15.5 (±7.0)	17.7 (±7.8)	20.1 (±8.0)	19.7 (±5.6)	16.1 (±7.6)
No. of H-ND smokers	41	202	441	613	227	64	1,588
CPD (±SD)	14.2 (±4.8)	17.6 (±6.8)	20.7 (±8.9)	24.9 (±10.9)	26.7 (±12.4)	25.0 (±8.4)	22.8 (±10.5)

Supplemental Table 2: The influence of social-environmental factors on the smoking status of female adults

Characteristics	No. of Non-smokers	No. of Current Smokers	Unadjusted OR (95% CI)	P-value	Adjusted OR** (95% CI)	P-value
Parents Smoking:						
No	898	15	1		1	
Yes	2,413	93	2.31 (1.33-4.00)	0.003	1.86 (1.05-3.31)	0.03
Number of smokers living in household:						
0	761	16	1		1	
1	1,563	47	1.43 (0.81-2.54)	0.22	1.33 (0.72-2.45)	0.36
≥ 2	1,055	49	2.21 (1.25-3.91)	0.007	1.49 (0.81-2.76)	0.20
Percent of smoke friends:						
≤ 25%	2,545	62	1		1	
26 – 50%	554	33	2.45 (1.59-3.77)	<0.0001	2.97 (1.85-4.76)	<0.0001
51-100%	255	18	2.90 (1.69-4.97)	0.0001	4.89 (2.70-8.89)	<0.0001

**: Adjusted for age, martial status, educational level, annual family income and BMI, as well as social-environmental factors by using multiple logistic regression models.
